# Serum Metabolite Biomarkers for Pancreatic Tumors: Neuroendocrine and Pancreatic Ductal Adenocarcinomas—A Preliminary Study

**DOI:** 10.3390/cancers15123242

**Published:** 2023-06-19

**Authors:** Karolina Skubisz, Krzysztof Dąbkowski, Emilia Samborowska, Teresa Starzyńska, Anna Deskur, Filip Ambrozkiewicz, Jakub Karczmarski, Mariusz Radkiewicz, Katarzyna Kusnierz, Beata Kos-Kudła, Tadeusz Sulikowski, Patrycja Cybula, Agnieszka Paziewska

**Affiliations:** 1 Institute of Health Sciences, Faculty of Medical and Health Sciences, Siedlce University of Natural Sciences and Humanities, 08-110 Siedlce, Poland; karolina.skubisz@gmail.com (K.S.); patrycja.cybula@uph.edu.pl (P.C.); 2 Department of Laboratory Diagnostics and Clinical Immunology of Developmental Age, Pediatric Hospital of Medical University of Warsaw, 02-091 Warsaw, Poland; 3 Department of Gastroenterology, Pomeranian Medical University in Szczecin, 70-204 Szczecin, Poland; dabkowskikrzysztof@wp.pl (K.D.); anndes@wp.pl (A.D.); 4 Mass Spectrometry Laboratory, Institute of Biochemistry and Biophysics, Polish Academy of Sciences, 02-106 Warsaw, Poland; sambor@ibb.waw.pl (E.S.); pocztakuby@gmail.com (J.K.); m.radkiewicz@ibb.waw.pl (M.R.); 5 Laboratory of Translational Cancer Genomics, Biomedical Center, Faculty of Medicine in Pilsen, Charles University, Alej Svobody 1665/76, 32300 Pilsen, Czech Republic; filip.ambrozkiewicz@lfp.cuni.cz; 6 The Department of Gastrointestinal Surgery, Medical University of Silesia, 40-752 Katowice, Poland; kkusnierzchir@gmail.com; 7 Department of Endocrinology and Neuroendocrine Tumours, Department of Pathophysiology and Endocrinology, Medical University of Silesia, 40-752 Katowice, Poland; bkoskudla@sum.edu.pl; 8 Department of General, Minimally Invasive and Gastroenterological Surgery, Pomeranian Medical University in Szczecin, 70-204 Szczecin, Poland; sulikowskit@wp.pl; 9 Molecular Biology Laboratory, Department of Diagnostic Hematology, Institute of Hematology and Transfusion Medicine, 02-776 Warsaw, Poland

**Keywords:** pancreatic ductal adenocarcinoma (PDAC), neuroendocrine pancreatic tumor (PNET), pancreas, pancreatic tumor, metabolite, metabolome, Biocrates, glutamine, serotonine, acylcarnitine, carnitine, acetylcarnitine, C2, AbsoluteIDQ^®^ p180 kit, glicerophospholipids, amino acids

## Abstract

**Simple Summary:**

Pancreatic cancer is a significant problem worldwide. Most cancers are diagnosed at an advanced stage. Limited knowledge of the pathogenesis of pancreatic tumors results in limited diagnostic and therapeutic possibilities. Using metabolome analysis, we identified unique metabolite profiles specific not only for pancreatic ductal adenocarcinoma (PDAC) but also for neuroendocrine pancreatic tumor (PNET), which may be helpful to understand the pathogenesis of pancreatic diseases. Additionally, we discovered that disturbed metabolites, mainly acetylcarnitine C2, serotonin, and glycerophospholipid PC aa C34:1, have potential to be used as biomarkers for diagnosing and monitoring the progression of pancreatic tumors. Serum-circulating metabolites can be easily monitored without invasive procedures; they show the current condition of clinical patients and therefore help with pharmacological treatments or dietary strategies.

**Abstract:**

Background: Pancreatic cancer is the most common pancreatic solid malignancy with an aggressive clinical course and low survival rate. There are a limited number of reliable prognostic biomarkers and a need to understand the pathogenesis of pancreatic tumors; neuroendocrine (PNET) and pancreatic ductal adenocarcinomas (PDAC) encouraged us to analyze the serum metabolome of pancreatic tumors and disturbances in the metabolism of PDAC and PNET. Methods: Using the AbsoluteIDQ^®^ p180 kit (Biocrates Life Sciences AG, Innsbruck, Austria) with liquid chromatography–mass spectrometry (LC-MS), we identified changes in metabolite profiles and disrupted metabolic pathways serum of NET and PDAC patients. Results: The concentration of six metabolites showed statistically significant differences between the control group and PDAC patients (*p.adj* < 0.05). Glutamine (Gln), acetylcarnitine (C2), and citrulline (Cit) presented a lower concentration in the serum of PDAC patients, while phosphatidylcholine aa C32:0 (PC aa C32:0), sphingomyelin C26:1 (SM C26:1), and glutamic acid (Glu) achieved higher concentrations compared to serum samples from healthy individuals. Five of the tested metabolites: C2 (FC = 8.67), and serotonin (FC = 2.68) reached higher concentration values in the PNET serum samples compared to PDAC, while phosphatidylcholine aa C34:1 (PC aa C34:1) (FC = −1.46 (0.68)) had a higher concentration in the PDAC samples. The area under the curves (AUC) of the receiver operating characteristic (ROC) curves presented diagnostic power to discriminate pancreatic tumor patients, which were highest for acylcarnitines: C2 with AUC = 0.93, serotonin with AUC = 0.85, and PC aa C34:1 with AUC = 0.86. Conclusions: The observations presented provide better insight into the metabolism of pancreatic tumors, and improve the diagnosis and classification of tumors. Serum-circulating metabolites can be easily monitored without invasive procedures and show the present clinical patients’ condition, helping with pharmacological treatment or dietary strategies.

## 1. Introduction

Pancreatic cancer (PC) is the most common pancreatic solid malignancy, characterized by an aggressive clinical course and survival rate that does not exceed 5% [1]. Most tumors are diagnosed at advanced stages [2]. Unfortunately, the therapeutic options for PC are quite limited due to the relatively late diagnosis and resistant nature of the tumor [3]. The only curative treatment is based on extensive resections that permanently change the anatomy and physiology of the digestive tract. Moreover, even after the radical surgery, the disease tends to recur, so long-term survivals are rare. Therapeutic efficacy is also not satisfactory due to the limited number of reliable prognostic biomarkers and limitation with knowledge about the pathogenesis of pancreatic tumors [4]. Both cancer and treatment are associated with metabolic and nutritional disorders: severe malnutrition, type 3c diabetes and exocrine pancreatic insufficiency [5]. There is no effective screening test for malignancy. The most frequently measured biomarker in PC is CA19-9 (carbohydrate antigen); however, due to its low sensitivity (59–64%), it is insufficient to detect the disease at an early stage of development [6,7].

Pancreatic neuroendocrine tumors (PNETs) originate from the endocrine pancreatic cells and constitute about 2% of neoplasms of this gland. Contrary to pancreatic cancer, these tumors have hormonal activity, which in case of so-called functioning tumors can lead to specific clinical symptoms (e.g., in the case of insulinoma and gastrinoma). Different tumor biology, origin, overall better prognosis and patient condition at the time of diagnosis make PNETs cases valuable for comparative analysis.

While changes occurring in the genome, transcriptome, or proteome may determine the predisposition to specific biological processes, changes in the metabolome reflect the current physiological state of the cell, tissue, organ, and the whole organism [8]. The development of cancer cells is associated with disturbed metabolism [9]. The pancreas is a crucial organ that regulates metabolism. In functional terms, the pancreas consists of the endocrine part—responsible for the production of hormones, which are linked to the regulation of carbohydrate metabolism and exocrine–digestive, producing pancreatic juice containing digestive enzymes, which supports the metabolism of nutrients [10].

Using an AbsoluteIDQ^®^ p180 kit (Biocrates Life Sciences AG) with liquid chromatography–mass spectrometry (LC-MS), was pivotal to identifying changes in metabolite profiles and disrupted metabolic pathways for a better understanding of tumorigenesis in PNETs and PDACs.

Results obtained in the course of this project present significantly different metabolites, unique to PDAC and PNET. It creates new opportunities in the search for biomarkers, therapeutic targets and the use of metabolic disturbances as prognostic factors in response to the treatment of PDAC and PNET.

## 2. Materials and Methods

### 2.1. Patients and Study Design

This study involved patients with pancreatic ductal adenocarcinomas (PDACs) and nonfunctioning pancreatic neuroendocrine tumors (PNETs), hospitalized in the Department of Gastroenterology, Pomeranian Medical University, and the Department of Gastrointestinal Surgery, Medical University of Silesia, Katowice, Poland. The study was approved by the Bioethics Committee of the Pomeranian Medical University kB-0012/32/14, dated 17 March 2014, and the Bioethics Committee of the Medical University of Silesia KNW/0022/KB1/102/II/17/19. Written informed consent was obtained from all patients. 

A study was performed with serum samples from patients diagnosed with PDAC (*n* = 15, age range 39–84, median 61, male/female—8/7), PNETs (*n* = 16, age range 29–81, median 61.5, male/female—8/8) and control group (*n* = 10, age range 41–83, median 60, male/female—6/4). The patient characteristics are presented in Table 1. Each patient with PDAC and PNET underwent staging with abdominal CT and chest X-ray, the diagnosis was made by a histopathological assessment of the specimen obtained by biopsy (endoscopic ultrasound guided, percutaneous, or obtained surgically).

The patients with pancreatic cancer and neuroendocrine tumors were recruited retrospectively from our computer database. The blood sample was collected in the moment of disease presentation (first hospital stay due to disease). The patients were in good clinical condition.

Recruitment criteria.

We excluded patients with a history of chemotherapy and cancers other than those analyzed and patients from clinical trials who were taking drugs/placebo (due to the unknown effect of the experimental treatment used).

We included patients with a diagnosis of adenocarcinoma or pancreatic NETs confirmed by histopathological examination and in good general condition with no significant comorbidities.

In the study group of patients, 8 had arterial hypertension, 3 had ischemic heart disease, and 1 had a history of epilepsy. In order to obtain a possibly homogeneous study group, patients with chronic systemic, hematological, and autoimmune diseases; chronic obstructive pulmonary disease; uncontrolled diabetes; and kidney and thyroid diseases were excluded from among patients operated on due to adenocarcinoma and PNET.

The characteristics of the PDAC and PNET patients are presented in Table 1.

### 2.2. Quantification of Serum Metabolites

#### 2.2.1. Chemicals

LC-MS-grade acetonitrile, HPLC-grade ethanol, HPLC-grade methanol, and formic acid were purchased from J.T. Baker (Phillipsburg, NJ, USA). Pyridine and phenyl isothiocyanate (PITC) were obtained from Merck Life Science (Darmstadt, Germany). Ultrapure water (Milli-Q water) was produced by using a water purification system (Milli-Q, Millipore, Milford, MA, USA). The AbsoluteIDQ^®^ p180 kit was obtained from Biocrates Life Sciences AG (Innsbruck, Austria). With the AbsoluteIDQ^®^ p180 kit, we analyzed 188 metabolites: amino acids (21), biogenic amines (21), monosaccharides (1), lipids (acylcarnitines (40), glycerophospholipids (90), and sphingomyelins (15)).

#### 2.2.2. Sample Preparation

Metabolites were analyzed according to protocol “User Manual, AbsoluteIDQ^®^ p180 kit—Waters Edition”. Serum samples were stored at −80 °C and before analysis were thawed, centrifuged at 2750× *g*, 4 °C for 5 min, and then mixed at 1200 RPM for 15 min.

A 10 µL of internal standard (IS) and 10 µL of the sample were added to the assigned well in a 96-well plate. All samples were dried under a nitrogen stream using a Positive Pressure-96 Processor for 30 min and derivatized for 25 min at room temperature using 50 µL of derivatization mixture. Next, the mixture was dried using a positive pressure manifold for 60 min, and 300 µL of extraction solvent was added, vortexed at 450 RPM for 30 min, and centrifuged at 500× *g* for 2 min to elute the metabolites. A 150 µL of the eluted extract was transferred to a 96-well LC plate, diluted with 150 µL pure water, and 2 µL was injected. A 10 µL of the mixture was transferred to a 96-well FIA (flow-injection analysis) plate and diluted with 490 µL of FIA solvent and injected in FIA mode.

#### 2.2.3. LC-MS Analyses

A Waters Acquity Ultra Performance Liquid Chromatograph coupled with a Waters TQ-S triple-quadrupole mass spectrometer was used during this study. Waters MassLynx software V4.2 SCN1035 was used for the instrument control and data acquisition, and Waters TargetLynx was used to process the data. Chromatographic separation of amino acids and biogenic amines was achieved using a Waters BEH C18 column (1.7 µm, 2.1 mm × 50 mm) and Waters BEH C18 guard column (1.7 µm, 2.1 mm × 5 mm). Analysis was performed in MRM mode with positive electrospray ionization. The FIA extract was analyzed in positive mode to capture acylcarnitines, glycerophospholipids, and sphingolipids, while hexoses were monitored in negative ionization mode. Concentrations of all analytes were calculated using MetIDQ™ software Oxygen DB110-3005.

### 2.3. Statistical Analysis

At the beginning of the analysis, metabolite concentration values that were below the limit of detection (LOD) or the limit of quantification (LOQ) were, respectively, replaced by half of the LOD and half of from the LOD to LOQ value. For concentrations above the upper limit of quantification (ULOQ), the ULOQ values increased by one were assumed.

Statistical analysis was performed in R (version 4.2.2). Significant differences were determined by Wilcoxon signed rank test. Metabolite was considered significant when the corrected *p-value* < 0.05. Correlation between metabolites and clinical parameters were assessed by Spearman correlation. To assess the impact of PNETs localization, we performed the analysis using the Kruskal Wallis test (without correction) to select differentiating metabolites and then the Wilcoxon test was performed for paired observations (comparing metabolite concentrations depending on tumor location) with Benjamini Hochberg’s correction.

Figures were prepared using R (version 4.1.0). Violin plots were made using the ggpubr package (version 0.4.0) and receiver operating characteristic (ROC) curves were constructed using the pROC package (version 1.18.0).

Metabolite set enrichment analysis (MSEA) and figures presenting the results of these analyzes were made using the online MetaboAnalyst 5.0 platform. The KEGG database with 84 metabolite sets based on human metabolic pathways was selected as the metabolite set library.

## 3. Results

### 3.1. Serum Metabolites Concentrations

We analyzed 154 metabolites (20 amino acids, 9 biogenic amines, 1 monosaccharide 25 acylcarnitines, 85 glycerophospholipids, and 14 sphingomyelins) that were detected in at least 40% of blood serum samples (Supplementary Materials Table S1).

### 3.2. Analysis of Metabolic Profiles

#### 3.2.1. Control vs. PDAC

The concentration of 23 metabolites showed statistically significant (*p-value* < 0.05) differences between the control group and the tested group with PDAC (Table 2). Six were statistically significant after applying the correction for multiple testing (*p.adj* < 0.05): Gln, PC aa C32:0, C2, SM C26:1, Cit, Glu. Glu, C2, and Cit presented a lower concentration in the serum of PDAC patients, while PC aa C32:0, SM C26:1, and Glu achieved higher concentrations compared to serum samples from healthy individuals.

#### 3.2.2. Control vs. PNET

We identified nine metabolites whose concentrations were statistically significantly different (*p-value* < 0.05) between the healthy and the PNET study groups (Table 3): PC ae C38:3, SM (OH) C22:1, SDMA, C14:1, PC ae C40:3, lysoPC a C20:3, PC aa C34:2, C14:2, and Cit. After correction for multiple testing, none of them reached statistical significance (*p.adj* < 0.05). All of the above statistically significant metabolites showed lower concentrations in tested samples with PNET, except for lysoPC a C20:3, which achieved higher concentrations for PNETs.

#### 3.2.3. PNET vs. PDAC

**Figure 1:**
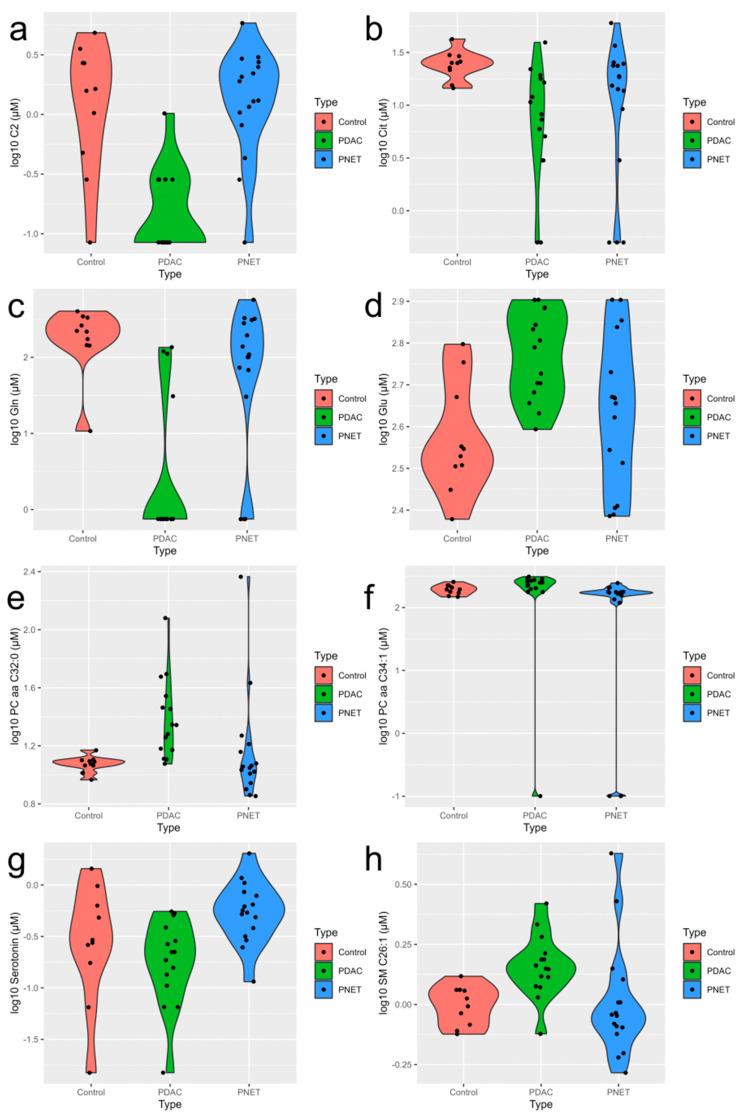
Violin plots for the selected metabolites significantly (*p.adj* < 0.05) different between the serum of pancreatic tumor patients and control serum samples (**a**) C2, (**b**) Cit, (**c**) Gln, (**d**) Glu, (**e**) PC aa C32:0, (**f**) PC aa C34:1, (**g**) serotonin, (**h**) SM C26:1. For each tested group, numeric values are represented as diamonds, the corresponding probability densities are represented as plain traits, and the mean and standard error are represented by black circles and segments, respectively (ggplot2 package, R).

As a result of the analyses, we identified the presence of 40 metabolites whose concentrations statistically significantly (*p-value* < 0.05) differentiate between PNETs and PDACs (Table 4, Figure 1).

Five of them achieved statistical significance after applying the correction for multiple testing (*p.adj* < 0.05): C2, PC aa C34:1, Serotonin, PC aa C32:0, PC ae C42:2. C2, serotonin, and PC ae C42:2 reached higher concentration values in the PNET serum samples, while PC aa C34:1 and PC aa C32:0 had a higher concentration in the PDAC samples.

### 3.3. Metabolic Pathway Analysis of Serum Metabolites

MetaboAnalyst was used to perform metabolite set enrichment analysis (MSEA) and determine pathways significantly enriched in PNETs and PDACs samples using HMDB numbers as metabolite IDs, without data transformation, using the SMPDB database. 

**Figure 2:**
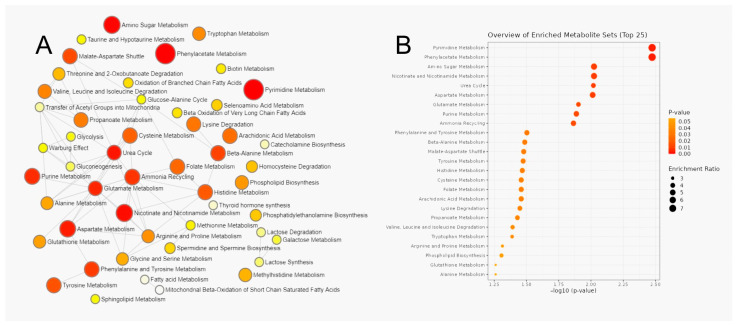
Metabolite set enrichment analysis (MSEA) compared between PNET and PDAC serum samples: (**A**) Interactive network of different metabolic pathways enriched in serum of tumor pancreatic patients. (**B**) The most enriched metabolic pathways in serum of tumor pancreatic patients. With the increase in color intensity (color close to red), the statistical significance increases, while the larger the diameter of the dots means a greater impact on the pathway.

**Figure 3:**
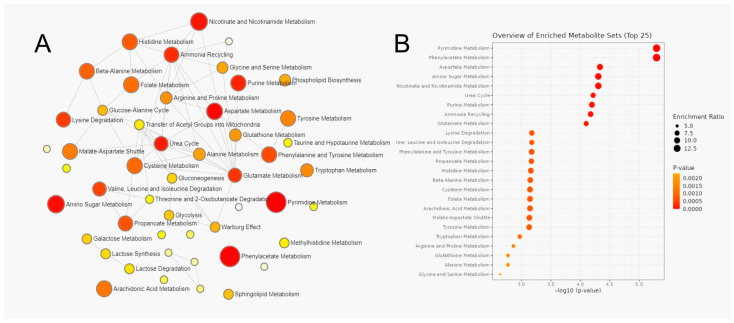
Metabolite set enrichment analysis (MSEA) compared between controls and PDAC serum samples: (**A**) Interactive network of different metabolic pathways enriched in serum of healthy individuals and PDAC. (**B**) The most enriched metabolic pathways in serum of tumor pancreatic patients. With the increase in color intensity (color close to red), the statistical significance increases, while the larger the diameter of the dots means a greater impact on the pathway.

The results of analysis are listed below (Table 5) and shown in Figure 2 and Figure 3.

In addition, lipid analysis showed that branched chain fatty acid oxidation, beta-oxidation of very long chain fatty acids, sphingolipid metabolism, phospholipid biosynthetic pathways are significantly (*p.adj* < 0.05) different between controls and PDAC. 

### 3.4. Receiver Operating Characteristic Curve Analysis for Specific Metabolites

**Figure 4:**
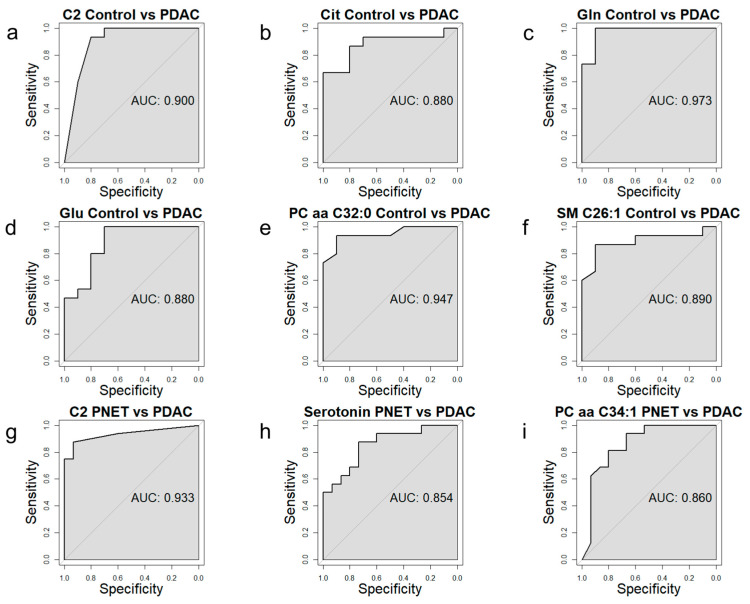
ROC curves presenting significantly (*p.adj* < 0.05) different metabolites between the serum of pancreatic tumor patients and the control serum samples. Controls vs. PDAC (**a**–**f**) and PNETs vs. PDAC (**g**–**i**).

Receiver operating characteristic (ROC) curves were generated for statistically significant metabolites (*p.adj* < 0.05) (Figure 4). The area under the curve (AUC) of the ROC curve presenting diagnostic power to discriminate PDAC patients from controls was highest for acylcarnitine: C2 with AUC = 0.90, amino acids: Gln with AUC = 0.97, Glu with AUC = 0.88, and Cit with AUC = 0.88; and lipids: PC aa C32:0 with AUC = 0.95 and SM C26:1 with AUC = 0.89.

Diagnostic power to discriminate PDAC patients from PNET presented very good value for acylcarnitines: C2 with AUC = 0.93; biogenic amines: Serotonin with AUC = 0.85 and glycerophospholipids: PC aa C34:1 with AUC = 0.86.

### 3.5. Correlation Analysis

Spearman correlation (r_s_) was performed for each clinical parameter of PDAC and PNET patients to discover their correlations with metabolite concentrations (Table 6). Glycerophospholipids were disturbed mainly according to the CA19-9 marker and stage of diagnosis for PDAC patients, and acylcarnitines; C3-DC C4-OH (r_s_ = 0.54), C4:1 (r_s_ = 0.55), C14:2 (r_s_ = 0.63), C16-OH (r_s_ = 0.58) according to metastasis; CRP (C18:1 (r_s_ = 0.59); CA 19-9; C16-OH (r_s_ = 0.71); and stage of diagnosis C14:1 (r_s_ = 0.61).

For PNET patients, the level of glycerophospholipids significantly correlate with CRP and stage of diagnosis, and acylcarnitines according to metastasis: C3-DC C4-OH (r_s_ = 0.56), and also stage of diagnosis C2 (r_s_ = 0.61), C3-DC (C4 OH) (r_s_ = 0.56, C14:2-OH (r_s_ = 0.62). Additionally, lysoPC a C18:1, PC ae C38:3 are associated with localization of PNETs. 

Amino acids were found to affect tumor patients with metastasis: Phe (r_s_ = 0.59) with PDAC, while Asn (r_s_ = 0,63), Gly (r_s_ = 0.55), for PNET patients. 

Additionally, Gly, Ser, Lys, and Leu were found to affect PNET patients according to localization. Ala (r_s_ = −0.54) and Phe (r_s_ = 0.59) correlated with CRP among PDAC patients and Asn (r_s_ = 0.69) for PNETs. 

The levels of several amines we detected in PNET serum were associated: Met SO (r_s_ = 0.92) with Ca 19-9, t4-OH-Pro (r_s_ = 0.64), and Kynurenine (r_s_ = −0.56) with stage. For PDAC only t4-OH-Pro (r_s_ = −0.55) was dependent on the CRP marker.

## 4. Discussion

PDAC is associated with a high mortality rate. It is frequently diagnosed at an advanced stage, even with distant metastases. A poor prognosis and low survival rate are observed. Not only the absence of typical symptoms at an early cancer stage is a significant problem, but the limited number of biomarkers and ineffectiveness of therapy are also profound disadvantages.

Understanding of the pancreatic cancer pathogenesis mechanism, related to disturbances in metabolism, is crucial to improve the diagnosis and the effectiveness of therapy. Cancerogenesis and progression of pancreatic cancer are linked to reprogramming observed in glucose and amino acids, and also in lipid metabolism [11].

The significant changes in the concentration of metabolites that we noticed in our study were discovered in previously published studies and were related to the malignant mechanism of pancreatic cancer and connected with cancer progression.

Neuroendocrine neoplasms of the pancreas analyzed in our project are rare tumors (5% of all cases), but recently more commonly diagnosed. They develop from cells of the diffuse endocrine system (DES) of the gastrointestinal tract. PNETs are less aggressive, generally benign, and slow-growing [12]. Early detection of pancreatic lesions and proper classification following progression and metastases are pivotal.

Chromogranin A (CgA) is a nonspecific biomarker in the NET management. Meta-analysis of chromogranin A level shows that it can be used to monitor disease, progression, recurrence, and response to treatment with sensitivity (46–100%) and specificity (68–90%), and an overall accuracy of 84%. However, it was concluded that circulating CgA is better for monitoring NET progression rather than its diagnosis [13]. 

NETest is a recently developed blood biomarker test where the expression profile of selected gene transcripts characteristic for NETs is analyzed. Comparison of the clinical utility of NETest and circulating CgA showed a significant advantage of the molecular biomarkers in the diagnosis and monitoring of NETs [14,15].

During the disease progression, PNETs become more aggressive; what is observed is an increase in the Ki-67 labeling index for proliferation assessment in biopsy tissue [16]. Additionally, the assessment of the proliferative marker (percentage of Ki67-positive cells) can therefore help the clinician in the proper diagnosis and sometimes in the selection of therapy [17,18]. New specific and sensitive biomarkers are needed to differentiate and classify pancreatic tumors. Notwithstanding the improvement in diagnostics, it is difficult to explain the mechanism of pathogenesis and discover the pathways implicated in advanced NENs and responsible for more aggressive biological phenotype. There are insufficient clinical data or studies to explain these pathological mechanisms implicated in the progression and presenting more aggressive character of the tumor compared to the initial stage [16]. 

Metabolomics, a new high-throughput approach that we adopted for our research is a strategy that can be used for both the early detection and the progression monitoring of the pancreatic tumors.

We hope that the metabolite markers will help to improve clinical diagnosis, differentiation of pancreatic tumors, and patient care.

Identifying the metabolite profiles specific and unique to pancreatic tumors (PDAC and PNET) and understanding the metabolism reprogramming in the pathogenesis process were significant for us and the aim of this study.

Our results show that metabolites significantly differ among not only serum samples from pancreatic tumors and healthy individuals, but also represent unique profiles for PDAC and PNET patients. 

Acylcarnitines are one of the analyzed groups of metabolites discovered in our study that significantly vary among pancreatic patients. Acylcarnitines are fatty acid metabolites playing an important role in cellular energy metabolism pathways as markers of energy metabolism, deficits in mitochondrial and peroxisomal β-oxidation activity, and insulin resistance. They are connected with metabolic disorders, cardiovascular diseases, and cancers, or can be disturbed as a result of dietary interventions [19,20]. Alterations in carnitine concentrations are related to the β-oxidation of fatty acids since carnitine serves as a shuttle to transport activated fatty acids (Acyl-CoA) from the cytosol into the mitochondrial matrix. It was shown that the extensive accumulation of acylcarnitine, present in obesity-driven HCC tissues, results in hepatocarcinogenesis [21]. Disturbances are seen in insulin sensitivity and in inflammation [22,23].

Hypocarnitinemia occurs in cachectic patients, pointing out that supplementation of carnitine deficiency is beneficial for patients [21,24,25]. Carnitines were also found as decreased in senescent PANC-1 cells and linked with a decrease in energy metabolism and mitochondrial dysfunction in senescent PANC-1 cells [19]. Nevertheless, there are still few publications describing the role of carnitines in pancreatic tumors and analysis of their correlations with clinical parameters of patients as a CRP marker or CA19-9.

Our metabolomic results present a different level of acylcarnitines in pancreatic serum samples from PDAC and PNET. Seven of them are disturbed in cancer samples, five (C3-DC (C4-OH), C14:2, C16-OH, C18:1, C18:2) show higher concentration in PDAC and two (C2, C16-OH) are lower, compared to PNETs and one (C2) to control serum samples. Acylcarnitine C2 not only differentiates the tumors group with significant fold change 8.84, but additionally C2 presents very good diagnostic power (0.9). There are limited data that link pancreatic tumors with carnitines.

We found that acylcarnitines significantly correlate with clinical parameters of PDAC patients: metastasis (C3-DC(C4-OH), C4:1, C14:2, C16-OH), CRP (C18:1), CA19-9 (C16-OH) and stage (C14:1), while in PNETs with: metastasis (C3-DC(C4-OH)) and stage (C2, C3-DC(C4-OH), C14:2-OH).

According to reports in the literature, supplementation with L-carnitine led to an increase in body mass index and an increase in overall survival in advanced pancreatic cancer patients [26]. Lower levels of carnitines were found among metabolites significantly changed during the perioperative period in patients diagnosed with pancreatic (pre-) malignancy and subjected to elective resection surgery under general anesthesia [27]. Additionally, the dexamethasone treatment results in higher carnitine levels compared to patients who did not receive dexamethasone [27].

Additionally, we confirmed that amino acids play roles in pancreatic tumor metabolism as potential markers differentiating pancreatic carcinomas from PNETs and healthy individuals’ sera. Among five disturbed amino acids (Asn, Cit, Gln, Glu, Phe), glutamine (*p.adj* = 0.0067; FC = 8.37) was the most significantly decreased amino acid in cancer sera compared to the control, thus showing very good diagnostic power to discriminate control and PDAC sera (AUC = 0.9). The level of glutamine was similarly different between cancer and PNET serum samples (Gln, *p-value* = 0.00282; FC = 6.09). Two other amino acids Asn and Cit, were also discovered as reduced in PDAC compared to controls. However, higher concentrations of amino acids (Asp, Glu, Phe) were seen in serum of PDAC with analysis in NETs serum and controls (Glu, Phe). Our results confirm the previously observed amino acid level abnormalities in the serum of pancreatic patients. The data obtained are related to disrupted metabolism, which is one of the hallmarks of tumor cells. Glutamine is an important metabolic substrate in cancer development and tumor metabolism and linked to abnormally high glutamine flux and overexpressed glutamine transporters [28,29]. Glutamine plays an important role in energy metabolism, inflammatory reactions, and immune processes in patients with severe acute pancreatitis (SAP) [30]. Additionally, cancer cells show increased glutamine uptake needed for proliferation, growth, and aerobic glycolysis (Warburg effect) [31,32,33]. Changes in the concentration of the amino acid (glutamine) influence and correlate with the severity of the disease [34].

Summarized results from a meta-analysis of 30 randomized controlled trials (RCTs) and a total of 1201 patients conclude that Gln supplementation is beneficial for SAP patients: improving the prognosis of patients; decreasing mortality (OR = 0.38, 95% CI: 0.21–0.69, *p* = 0.001); shortening total hospital stay; and decreasing adverse symptoms (OR = 0.45, *p* < 0.0001). In addition, there is improved liver, kidney, and immune function, compared with conventional nutrition [35,36] or with acute pancreatitis [37]. Adding glutamine to therapy significantly improves the efficacy of imipenem in the treatment of severe acute pancreatitis with abdominal infection (odds ratio = 0.78, 95% CI 0.71–0.86, *p* = 0.040) [38].

Oncolometabolic studies showed changes in the concentrations of potential diagnostic biomarkers. Glutamic acid and histidine were reported in seven studies, and glutamine and isoleucine in five studies, as correlated with the diagnostic area under the curve ranging from 0.68 to 1.00 (sensitivity: 0.43–1.00, specificity: 0.73–1.00) [39]. L-glutamine, and glutamic acid were found among serum metabolites using reversed-phase liquid chromatography (RPLC) and hydrophilic interaction liquid chromatography (HILIC) as potential markers to differentiate pancreatic carcinoma from benign disease (BD).

Amino acids (L-glutamine, glutamic acid, L-phenylalanine, L-tryptophan, and L-arginine) were identified in serum samples, discriminating pancreatic cancer, benign disease, and normal control, with sensitivity and specificity. They provide a sensitive, blood-borne diagnostic signature for the presence of cancer or its precursor lesions [40].

Serotonin, the only one of the tested biogenic amines, shows a significantly higher level (FC = 2.68; *p.adj* = 0.043) in PNET serum compared PDAC with a very good AUC = 0.854 discrimination among pancreatic tumor patients. The increased serotonin level seen in our results for PNETs is consistent with other studies and publications. This biogenic amine (5-HT) is a neurotransmitter identified as related to affecting emotion, behavior, sleep, health, pain, and cognition [41,42,43]. Moreover, serotonin is connected with the regulation of intestinal motility, vasoconstriction, amplification of platelet aggregation, and wound healing [44]. However, this higher concentration could influence the risk of heart failure in NET patients. Additionally, relative serotonin level measured with concentration of its urinary 5-hydroxyindoleacetic acid (u5-HIAA) is predictive of 1-year all-cause mortality in patients with NETs [45,46]. Serotonin plays a crucial role in tumor development, and impacts their growth and progression [44]. However, the role of serotonin on tumor growth is still unclear and complicated, whether it promotes or suppresses tumorigenesis [46]. With dose-dependent influence, it sometimes results in opposing effects on tumor growth; higher doses result in mitogenic effects and promote proliferation and, at lower doses, reduce tumor growth action on tumor vasculature. The role of serotonin and 5-HT receptor subtypes in cell proliferation is connected with angiogenesis, invasion, migration, and metastasis. Expression of serotonin receptors may be tissue-specific and dysregulated in human cancers. Serotonin at physiological levels functions as a potent angiokinesis and regulator of the angiogenesis of tumors. It influences the arterioles feeding the tumor by interaction with 5-HT1B and 2A receptors on vascular smooth muscles, while serotonin mediated vasodilation is due to its interaction with the 5-HT2B receptor present on endothelial cells. Serotonin influences and controls the immune system, and affects cytokine release from macrophages and monocytes. With complex interactions, it plays a role in inflammation and gut inflammation. Serotonin functions as a neurotransmitter that links inflammation and cancer development, resulting in the immune response during cancer progression. Preoperative serotonin correlates with progression-free survival and overall survival of neuroendocrine tumors [47].

Our results are consistent with available data, exploring the role of lipids in pancreatic diseases. In our study, we confirmed the dysregulation of glycerophospholipids and their role in the development of pancreatic cancer [48,49]. Ten glycerophospholipids were significantly different (two lysoPC): four present higher concentrations in PDAC and six in controls. Four glycerophospolipids (lysoPC a C20:3; PC aa C34:2; PC ae C38:3; PC ae C40:3) were lower in PNET serum, compared to control serum. Only lysoPC presented a higher concentration in PNET serum. Analysis of pancreatic tumor serum profiles revealed 20 different glycerophospolipids: 4 LysoPC, 11 higher in NET serum samples, and 5 in PDACs serum samples. Analysis of the sphingolipid content reported four (SM C16:0; SM C18:0; SM C24:1; SM C26:1) to be significantly higher in PDAC than in the control samples, and SM (OH) C22:1 was higher in serum compared to NETS. PNET and PDAC metabolomic profiles analysis revealed seven of the sphingomyelin at higher concentration in PDAC: SM C16:0, SM C16:1, SM C18:0, SM C18:1, SM C20:2, SM C24:1, and SM C26:1.

It was reported that lipids showed higher differentiating efficacy between PDAC and chronic pancreatitis (CP) (*p-value* < 0.0001) with a discriminating power AUC of 0.86 (95% CI 0.81–0.91, *p* < 0.0001) for all the altered metabolites (*n* = 88). Pathway enrichment analysis indicated sphingomyelin metabolism (impact value 0.29, FDR of 0.45) and TCA cycle (impact value 0.18, FDR of 0.06) to be prominent pathways in differentiating PDAC from CP in the pathway enrichment analysis [50,51].

One of the hallmarks of pancreatic cancer is a dense desmoplastic stromastroma, which creates a natural barrier against oxygen, nutrients, and the immune system [52]. Thus, cells have developed a mechanism that alters canonical metabolic pathways to counteract starvation [53]. Typical for this kind of metabolic modification is a switch to anaerobic glycolysis. In our data, among the statistically significant pathways, we can observe a high number of pathways connected with amino acid metabolism and pyrimidine/purine metabolism. Amino acid metabolism is upregulated in many cancers; additionally, many cancers tend to be addicted to particular amino acids. Moreover, amino acids promote survival and proliferation of cancer cells under stress conditions (e.g., oxidative, nutritional) [54]. (3) Pyrimidine/ purine metabolic pathways are highly conserved among all living organisms. They are vital in maintaining basic organism functions (e.g., biosynthesis of nucleic acids), and also dysfunction in that these pathways are related to cancer progression [55]. Our research shows that there are significant differences between healthy control and PDAC patients in dysregulated pathways, but what is more interesting is that we found similar metabolic pathways in our PNET group that also show dysregulation, although achieving lower significance. This may be connected with different mechanisms responsible for cancer development and etiology.

Metabolites that we found to differentiate pancreatic tumors were additionally noted in PubMed database to be linked with other gastrointestinal malignancies: esophageal [56,57,58,59,60,61,62], stomach/gastric (GC) [63,64,65,66,67,68,69,70], liver/ hepatocellular carcinoma (HCC) [71,72,73,74,75,76,77,78,79,80,81,82,83,84,85,86,87], and colon/colorectal (CRC) [88,89,90,91,92,93,94,95,96,97,98,99,100,101] (Supplement Table S2). We noticed that there is still limited knowledge concerning metabolism disturbances linked to creatine and phosphatidylcholine, especially C2 and PC 34. 

A limitation of this study is the small number of samples and patients. In addition, there is no follow-up information and no data on patient outcome. The presented results are from a preliminary study that needs to be continued. More studies based on larger groups of patients should be carried out to reveal the best metabolites (with high specificity and sensitivity) that can be used for diagnosis and monitoring of tumor progression and patient outcome.

## 5. Conclusions

The observations presented provide better insight into the metabolism of pancreatic tumors and the ways to improve the classification and diagnosis of the tumors. Serum-circulating metabolites can be easily monitored without invasive procedures; they show the current condition of clinical patients and therefore help with pharmacological treatments or dietary strategies.

Patients with pancreatic tumors (PDAC and PNET) face disease progression related to metabolic disorders. Therefore, the discovery of metabolic biomarkers that monitor disease progression is of fundamental importance.

## Figures and Tables

**Table 1:** Characteristics of PDAC and PNET patients. Data are presented as median with interquartile range.

	PDAC	PNET
age (years)	66 (58.0–75.5)	62 (42.0–70.5)
weight (kg)	62 (50.0–71.5)	77 (67.5–86.0)
height (cm)	165.0 (158.0–171.0)	176 (171.2–181.0)
tumor size (mm)	40 (35–52.2)	20 (17–30)
stage at diagnosis, no		
T1/T2/T3/T4	1/2/1/9	7/2/3/1
N0/N1	10/3	12/1
M0/M1	12/1	9/4
localization:		
head	13	6
corpus	1	2
tail	0	6
other/unknown	1	2
metastasis no/%:		
yes	6/40%	5/31%
no	9/60%	11/69%
WBC (thou/uL)	7.0 (5.9–9.2)	6.6 (5.8–7.2)
RBC (mil/uL)	4.2 (3.8–4.6)	4.7 (4.5–4.9)
PLT (thou/uL)	228.5 (148.2–276.2)	211.0 (188.2–261.0)
CRP (mg/L)	29.4 (10.8–86.1)	1.8 (1.0–3.2)
CA19-9 (U/mL)	534.5 (165.0–2172.5)	3.3 (3.0–6.9)

**Table 2:** Metabolites significantly different (*p-value* < 0.05) between the serum of healthy individuals and PDAC patients.

Control vs. PDAC						
Class	Metabolite	Name	*p*-Value	*p.adj*	FC	Up ↑/Down ↓- Control/PDAC
Acylcarnitines	C2	Acetylcarnitine	6.0 × 10^4^	0.03	8.84	↑
Amino acids	Asn	Asparagine	0.038	0.29	1.50	↑
	Cit	Citrulline	1.7 × 10^3^	0.04	2.21	↑
	Gln	Glutamine	4.4 × 10^5^	6.7 × 10^3^	8.37	↑
	Glu	Glutamic Acid	1.7 × 10^3^	0.04	−1.56	↓
	Phe	Phenylalanine	0.013	0.21	−1.32	↓
Biogenic amines	SDMA	Symmetric dimethylarginine	0.016	0.25	1.33	↑
Glycerophospholipids	lysoPC a C16:0	Lysophosphatidylcholine a C16:0	0.020	0.25	−1.37	↓
	lysoPC a C18:1	Lysophosphatidylcholine a C18:1	0.033	0.28	−1.32	↓
	PC aa C32:0	Phosphatidylcholine aa C32:0	2.0 × 10^4^	1.7 × 10^2^	−2.56	↓
	PC aa C34:1	Phosphatidylcholine aa C34:1	0.046	0.32	−1.16	↓
	PC aa C42:0	Phosphatidylcholine aa C42:0	0.026	0.28	1.27	↑
	PC aa C42:1	Phosphatidylcholine aa C42:1	0.019	0.25	1.40	↑
	PC aa C42:2	Phosphatidylcholine aa C42:2	0.048	0.32	1.35	↑
	PC ae C40:1	Phosphatidylcholine ae C40:1	0.026	0.28	1.42	↑
	PC ae C42:2	Phosphatidylcholine ae C42:2	0.035	0.28	1.35	↑
	PC ae C42:3	Phosphatidylcholine ae C42:3	0.043	0.31	1.32	↑
Sphingolipids	SM (OH) C22:1	Hydroxysphingomyelin C22:1	0.013	0.21	1.32	↑
	SM C16:0	Sphingomyelin C16:0	0.031	0.28	−1.32	↓
	SM C18:0	Sphingomyelin C18:0	0.033	0.28	−1.52	↓
	SM C24:1	Sphingomyelin C24:1	0.004	0.09	−1.35	↓
	SM C26:1	Sphingomyelin C26:1	1.3 × 10^3^	0.04	−1.49	↓
Monosaccharides	H1	Hexoses	0.031	0.28	−1.49	↓

**Table 3:** Metabolites significantly different (*p-value* < 0.05) between the serum of healthy individuals and PNET patients.

Control vs. PNET						
Class	Metabolite	Name	*p*-Value	*p.adj*	FC	Up ↑/Down ↓- Control/PNET
Acylcarnitines	C14:1	Tetradecenoylcarnitine	0.029	0.72	1.21	↑
	C14:2	Tetradecadienylcarnitine	0.048	0.72	1.07	↑
Amino acids	Cit	Citrulline	0.048	0.72	1.40	↑
Biogenic amines	SDMA	Symmetric dimethylarginine	0.027	0.72	1.15	↑
Glycerophospholipids	lysoPC a C20:3	Lysophosphatidylcholine a C20:3	0.035	0.72	−1.43	↓
	PC aa C34:2	Phosphatidylcholine aa C34:2	0.040	0.72	1.16	↑
	PC ae C38:3	Phosphatidylcholine ae C38:3	0.020	0.72	1.06	↑
	PC ae C40:3	Phosphatidylcholine ae C40:3	0.029	0.72	1.12	↑
Sphingolipids	SM (OH) C22:1	Hydroxysphingomyelin C22:1	0.023	0.72	1.31	↑

**Table 4:** Metabolites significantly different (*p-value* < 0.05) between the serum of PNET patients and PDAC patients.

PNET vs. PDAC						
Class	Metabolite	Name	*p*-Value	*p.adj*	FC	Up/Down- PNET/PNET
Acylcarnitines	C2	Acetylcarnitine	3.0 × 10^5^	4.6 × 10^3^	8.67	↑
	C3-DC (C4-OH)	Malonylcarnitine	0.034	0.16	−1.09	↓
	C14:2	Tetradecadienylcarnitine	0.028	0.14	−1.14	↓
	C16-OH	Hydroxyhexadecanoylcarnitine	0.020	0.12	1.19	↑
	C16:2-OH	Hydroxyhexadecadienoylcarnitine	0.015	0.11	−1.01	↓
	C18:1	Octadecenoylcarnitine	0.014	0.11	−1.30	↓
	C18:2	Octadecadienylcarnitine	0.034	0.16	−1.01	↓
Amino acids	Asn	Asparagine	0.015	0.11	1.65	↑
	Asp	Aspartic acid	0.040	0.17	−1.43	↓
	Gln	Glutamine	2.8E−03	0.06	6.09	↑
	Glu	Glutamic Acid	0.044	0.18	−1.28	↓
	Phe	Phenylalanine	0.013	0.11	−1.33	↓
Biogenic amines	Serotonin	Serotonin	8.0 × 10^4^	0.04	2.68	↑
Glycerophospholipids	lysoPC a C16:0	Lysophosphatidylcholine a C16:0	3.2 × 10^3^	0.06	−1.37	↓
	lysoPC a C17:0	Lysophosphatidylcholine a C17:0	0.012	0.11	−1.19	↓
	lysoPC a C18:0	Lysophosphatidylcholine a C18:0	0.042	0.17	−1.39	↓
	lysoPC a C20:3	Lysophosphatidylcholine a C20:3	0.014	0.11	1.53	↑
	PC aa C32:0	Phosphatidylcholine aa C32:0	1.5 × 10^3^	0.05	−1.12	↓
	PC aa C34:1	Phosphatidylcholine aa C34:1	7.0 × 10^4^	0.04	−1.46	↓
	PC aa C34:2	Phosphatidylcholine aa C34:2	0.028	0.14	−1.20	↓
	PC aa C36:2	Phosphatidylcholine aa C36:2	0.028	0.14	−1.35	↓
	PC aa C36:6	Phosphatidylcholine aa C36:6	8.1 × 10^3^	0.09	1.90	↑
	PC aa C38:5	Phosphatidylcholine aa C38:5	0.046	0.18	1.85	↑
	PC aa C40:1	Phosphatidylcholine aa C40:1	0.049	0.19	1.32	↑
	PC aa C42:0	Phosphatidylcholine aa C42:0	0.031	0.15	1.30	↑
	PC aa C42:1	Phosphatidylcholine aa C42:1	6.4 × 10^3^	0.08	1.46	↑
	PC aa C42:2	Phosphatidylcholine aa C42:2	0.019	0.12	1.36	↑
	PC aa C42:6	Phosphatidylcholine aa C42:6	0.016	0.11	1.45	↑
	PC ae C36:0	Phosphatidylcholine ae C36:0	8.1 × 10^3^	0.09	−1.19	↓
	PC ae C40:1	Phosphatidylcholine ae C40:1	4.7 × 10^3^	0.07	1.51	↑
	PC ae C42:1	Phosphatidylcholine ae C42:1	0.040	0.17	1.34	↑
	PC ae C42:2	Phosphatidylcholine ae C42:2	1.8 × 10^3^	0.05	1.50	↑
	PC ae C42:3	Phosphatidylcholine ae C42:3	4.7 × 10^3^	0.07	1.33	↑
Sphingolipids	SM C16:0	Sphingomyelin C16:0	0.010	0.10	−1.23	↓
	SM C16:1	Sphingomyelin C16:1	0.026	0.14	−1.22	↓
	SM C18:0	Sphingomyelin C18:0	0.015	0.11	−1.33	↓
	SM C18:1	Sphingomyelin C18:1	0.030	0.15	−1.32	↓
	SM C20:2	Sphingomyelin C20:2	0.024	0.14	−1.43	↓
	SM C24:1	Sphingomyelin C24:1	2.2 × 10^3^	0.06	−1.79	↓
	SM C26:1	Sphingomyelin C26:1	5.6 × 10^3^	0.08	−1.23	↓

**Table 5:** (**a**) The most statistically significant differing metabolic pathways between PNET and PDAC samples. (**b**) The most statistically significant differing metabolic pathways between controls and PDAC samples.

(**a**)			
**Metabolic Pathway**	**Total. Cmpd**	**Hits**	***p*-Value**
Pyrimidine Metabolism	59	1	3.36 × 10^−^³
Phenylacetate Metabolism	9	1	3.36 × 10^−^³
Amino Sugar Metabolism	33	2	9.47 × 10^−^³
Nicotinate and Nicotinamide Metabolism	37	2	9.47 × 10^−^³
Urea Cycle	29	7	9.58 × 10^−^³
Aspartate Metabolism	35	6	9.70 × 10^−^³
Glutamate Metabolism	49	5	1.25 × 10^−2^
Purine Metabolism	74	4	1.30 × 10^−2^
Ammonia Recycling	32	7	1.37 × 10^−2^
Phenylalanine and Tyrosine Metabolism	28	3	3.14 × 10^−2^
Beta-Alanine Metabolism	34	3	3.26 × 10^−2^
Malate-Aspartate Shuttle	10	2	3.32 × 10^−2^
Tyrosine Metabolism	72	3	3.36 × 10^−2^
Histidine Metabolism	43	2	3.41 × 10^−2^
Cysteine Metabolism	26	1	3.47 × 10^−2^
Folate Metabolism	29	1	3.47 × 10^−2^
Arachidonic Acid Metabolism	69	2	3.47 × 10^−2^
Lysine Degradation	30	2	3.56 × 10^−2^
Propanoate Metabolism	42	2	3.72 × 10^−2^
Valine, Leucine, and Isoleucine Degradation	60	4	4.05 × 10^−2^
Tryptophan Metabolism	60	5	4.09 × 10^−2^
Arginine and Proline Metabolism	53	7	4.86 × 10^−2^
Phospholipid Biosynthesis	29	2	4.94 × 10^−2^
Glutathione Metabolism	21	3	5.48 × 10^−2^
Alanine Metabolism	17	3	5.48 × 10^−2^
(**b**)			
**Metabolic Pathway**	**Total. Cmpd**	**Hits**	***p*-Value**
Pyrimidine Metabolism	59	1	5.06 × 10^−6^
Phenylacetate Metabolism	9	1	5.06 × 10^−6^
Aspartate Metabolism	35	6	4.64 × 10^−5^
Amino Sugar Metabolism	33	2	4.95 × 10^−5^
Nicotinate and Nicotinamide Metabolism	37	2	4.95 × 10^−5^
Urea Cycle	29	7	6.06 × 10^−5^
Purine Metabolism	74	4	6.36 × 10^−5^
Ammonia Recycling	32	7	6.68 × 10^−5^
Glutamate Metabolism	49	5	7.95 × 10^−5^
Lysine Degradation	30	2	6.69 × 10^−5^
Valine, Leucine, and Isoleucine Degradation	60	4	6.69 × 10^−4^
Phenylalanine and Tyrosine Metabolism	28	3	6.72 × 10^−4^
Propanoate Metabolism	42	2	6.79 × 10^−4^
Histidine Metabolism	43	2	6.93 × 10^−4^
Beta-Alanine Metabolism	34	3	7.09 × 10^−4^
Cysteine Metabolism	26	1	7.17 × 10^−4^
Folate Metabolism	29	1	7.17 × 10^−4^
Arachidonic Acid Metabolism	69	2	7.17 × 10^−4^
Malate-Aspartate Shuttle	10	2	7.34 × 10^−4^
Tyrosine Metabolism	72	3	7.41 × 10^−4^
Tryptophan Metabolism	60	5	1.07 × 10^−3^
Arginine and Proline Metabolism	53	7	1.37 × 10^−3^
Glutathione Metabolism	21	3	1.70 × 10^−3^
Alanine Metabolism	17	3	1.70 × 10^−3^
Glycine and Serine Metabolism	59	8	2.29 × 10^−3^
Phospholipid Biosynthesis	29	2	1.70 × 10^−3^

**Table 6:** Analysis of metabolite and clinical parameter correlation.

Patients	Class	CRP		CA19-9		Stage (1/2/3/4)		Metastasis (yes/no)	
		Metabolite	Correlation Coefficient	Metabolite	Correlation Coefficient	Metabolite	Correlation Coefficient	Metabolite	Correlation Coefficient
PDAC	Glyceropfospholipids	PC ae C38:0	−0.6	lysoPC a C24:0	0.63	lysoPC a C24:0	0.57		
		PC ae C40:5	−0.56	lysoPC a C26:0	0.73	lysoPC a C26:0	0.62		
				lysoPC a C26:1	0.71	lysoPC a C28:0	0.59		
				lysoPC a C28:0	0.7	PC aa C24:0	0.58		
				lysoPC a C28:1	0.71	PC aa C36:0	0.57		
				PC aa C24:0	0.65	PC ae C40:3	0.58		
				PC aa C42:0	0.63				
				PC aa C42:1	0.68				
				PC ae C30:2	0.65				
				PC ae C42:2	0.61				
	Sphingolipids			SM OH C14:1	0.69	SM C18:0	0.6	SM OH C14:1	0.57
				SM OH C16:1	0.57				
	Acylcarnitines	C18:1	0.59	C16-OH	0.71	C14:1	0.61	C3-DC (C4 OH)	0.54
								C4:1	0.55
								C14:2	0.63
								C16-OH	0.58
	Amino acids	Ala	−0.54					Phe	0.63
		Phe	0.59						
	Biogenic amines	t4-OH-Pro	−0.55						
PNET	Glyceropfospholipids	lysoPC a C26:0	−0.84	PC aa C32:2	−0.83			PC aa C32:1	0.57
		lysoPC a C28:0	−0.88	PC aa C34:1	−0.79			PC aa C32:2	0.54
		lysoPC a C28:1	−0.72	PC aa C34:4	−0.9			PC ae C34:3	0.51
		PC aa C24:0	−0.84	PC aa C36:1	−0.81				
		PC ae C38:1	−0.71	PC aa C36:2	−0.75				
		PC ae C38:2	−0.85	PC aa C36:5	−0.83				
				PC aa C36:6	−0.86				
				PC aa C38:5	−0.81				
				PC aa C40:3	−0.76				
				PC aa C40:4	−0.81				
				PC aa C40:5	−0.83				
				PC aa C40:6	−0.86				
				PC aa C42:4	−0.71				
				PC ae C44:3	−0.74				
	Sphingolipids			SM OH C22:1	−0.76	SM C20:2	0.56		
				SM C24:0	−0.74	SM C26:0	0.59		
	Acylcarnitines					C2	0.61	C3-DC C4-OH	0.56
						C3-DC (C4 OH)	0.56		
						C14:2-OH	0.62		
	Amino acids	Asn	0.69	Orn	0.71	Asn Gly	0.63 0.55		
	Biogenic amines					Kynurenine	−0.56		
				Met SO	0.92	t4-OH-Pro	0.64		

## Data Availability

The data presented in this study are available in this article and Supplementary Material.

## Supplementary Materials

The following supporting information can be downloaded at: https://www.mdpi.com/article/10.3390/cancers15123242/s1, Table S1: Metabolites concentrations [µM] for serum samples; Table S2: Association of metabolites (C2, serotonin, phosphatidylocholine) with other gastrointestinal malignanices; esophageal, stomach/gastric (GC), liver/ hepatocellular carcinoma (HCC), colon/colorectal (CRC) PubMed. 2015–2023.

## Abbreviations

5-HT	5-hydroxytryptamine
Ala	alanine
Asn	asparagine
Asp	aspartic acid
AUC	area under the curves
BD	benign disease
C14:1	tetradecenoylcarnitine
C14:2	tetradecadienylcarnitine
C14:2-OH	hydroxytetradecadienylcarnitine
C16:2-OH	hydroxyhexadecadienoylcarnitine
C16-OH	hydroxyhexadecanoylcarnitine
C18:1	octadecenoylcarnitine
C18:2	octadecadienylcarnitine
C2	acetylcarnitine
C3-DC (C4-OH)	malonylcarnitine
CgA	chromogranin A
Cit	citrulline
DES	diffuse endocrine system
FIA	flow-injection analysis
Gln	glutamine
Glu	glutamic acid
Gly	glycine
H1	hexose
HILIC	hydrophilic interaction liquid chromatography
Ile	isoleucine
IS	internal standard
LC-MS	liquid chromatography–mass spectrometry
LOD	limit of detection
LOQ	limit of quantification
lysoPC a C16:0	lysophosphatidylcholine a C16:0
lysoPC a C17:0	lysophosphatidylcholine a C17:0
lysoPC a C18:0	lysophosphatidylcholine a C18:0
lysoPC a C18:1	lysophosphatidylcholine a C18:1
lysoPC a C18:2	lysophosphatidylcholine a C18:2
lysoPC a C20:3	lysophosphatidylcholine a C20:3
lysoPC a C24:0	lysophosphatidylcholine a C24:0
lysoPC a C26:0	lysophosphatidylcholine a C26:0
lysoPC a C26:1	lysophosphatidylcholine a C26:1
lysoPC a C28:0	lysophosphatidylcholine a C28:0
lysoPC a C28:1	lysophosphatidylcholine a C28:1
Met SO	methionine-sulfoxide
MSEA	metabolite set enrichment analysis
NETs	neuroendocrine tumors
PA	pancreatic cancer
PC aa C24:0	phosphatidylcholine aa C24:0
PC aa C32:0	phosphatidylcholine aa C32:0
PC aa C32:1	phosphatidylcholine aa C32:1
PC aa C32:2	phosphatidylcholine aa C32:2
PC aa C34:1	phosphatidylcholine aa C34:1
PC aa C34:2	phosphatidylcholine aa C34:2
PC aa C34:4	phosphatidylcholine aa C34:4
PC aa C36:0	phosphatidylcholine aa C36:0
PC aa C36:1	phosphatidylcholine aa C36:1
PC aa C36:2	phosphatidylcholine aa C36:2
PC aa C36:5	phosphatidylcholine aa C36:5
PC aa C36:6	phosphatidylcholine aa C36:6
PC aa C38:5	phosphatidylcholine aa C38:5
PC aa C40:1	phosphatidylcholine aa C40:1
PC aa C40:3	phosphatidylcholine aa C40:3
PC aa C40:4	phosphatidylcholine aa C40:4
PC aa C40:5	phosphatidylcholine aa C40:5
PC aa C40:6	phosphatidylcholine aa C40:6
PC aa C42:0	phosphatidylcholine aa C42:0
PC aa C42:1	phosphatidylcholine aa C42:1
PC aa C42:2	phosphatidylcholine aa C42:2
PC aa C42:4	phosphatidylcholine aa C42:4
PC aa C42:6	phosphatidylcholine aa C42:6
PC ae C30:2	phosphatidylcholine ae C30:2
PC ae C34:3	phosphatidylcholine ae C34:3
PC ae C36:0	phosphatidylcholine ae C36:0
PC ae C38:0	phosphatidylcholine ae C38:0
PC ae C38:1	phosphatidylcholine ae C38:1
PC ae C38:2	phosphatidylcholine ae C38:2
PC ae C38:3	phosphatidylcholine ae C38:3
PC ae C40:1	phosphatidylcholine ae C40:1
PC ae C40:3	phosphatidylcholine ae C40:3
PC ae C40:5	phosphatidylcholine ae C40:5
PC ae C42:1	phosphatidylcholine ae C42:1
PC ae C42:2	phosphatidylcholine ae C42:2
PC ae C42:2	phosphatidylcholine ae C42:2
PC ae C42:3	phosphatidylcholine ae C42:3
PC ae C44:3	phosphatidylcholine ae C44:3
PDAC	pancreatic ductal adenocarcinoma
Phe	phenylalanine
PITC	pyridine and phenyl isothiocyanate
PNET	neuroendocrine pancreatic tumor
RCTs	randomized controlled trials
ROC	receiver operating characteristic
RPLC	reversed-phase liquid chromatography
SAP	severe acute pancreatitis
SDMA	symmetric dimethylarginine
Serotonin	serotonin
SM (OH) C14:1	hydroxysphingomyelin C14:1
SM (OH) C16:1	hydroxysphingomyelin C16:1
SM (OH) C22:1	hydroxysphingomyelin C22:1
SM C16:0	sphingomyelin C16:0
SM C16:1	sphingomyelin C16:1
SM C18:0	sphingomyelin C18:0
SM C18:1	sphingomyelin C18:1
SM C20:2	sphingomyelin C20:2
SM C24:0	sphingomyelin C24:0
SM C24:1	sphingomyelin C24:1
SM C26:0	sphingomyelin C26:0
SM C26:1	sphingomyelin C26:1
t4-OH-Pro	trans-4-Hydroxyproline
u5-HIAA	urinary 5-hydroxyindoleacetic acid
ULOQ	upper limit of quantification
