# Supplementary material for: Serum Metabolite Biomarkers for Pancreatic Tumors: Neuroendocrine and Pancreatic Ductal Adenocarcinomas—A Preliminary Study
Source: Cancers (Basel). 2023 Jun 19;15(12):3242. doi: 10.3390/cancers15123242 (PMC10296343; doi:10.3390/cancers15123242)
Supplement: Supplementary file 1 [file cancers-15-03242-s001.zip › cancers-2374543-supplementary Table S2.pdf]

Supplement Table S2. Association of metabolites (C2, serotonin, phosphatidylocholine) with other gastrointestinal malignancies; esophageal, stomach/gastric (GC), liver/ hepatocellular carcinoma (HCC), colon/colorectal (CRC) PubMed. 2015-2023

cancer	C2	serotonin	Phosphatidylcholine PC aa C34:1

esophageal squamous cell carcinoma (ESCC)	down-regulated carnitines in ESCC patients.<br>PMID: 29631075		de novo synthesis of phosphatidylcholine (PC) in esophageal cancer MCTS [56]<br><br>altered FA profiles (phosphatidylcholine) in plasma of the patients with squamous EC during neo-adjuvant chemoradiotherapy<br><br>abnormal FA metabolism in cancer; altered synthesis de novo, b-oxidation, desaturation, and elongation [57]<br><br>metabolomic alterations associated with ILF2 and ILF3 in ESCC tissues<br><br>enriched phosphatidylcholine biosynthesis, fatty acid metabolism pathways, involved in the oxidation of fatty acids [58]<br><br>enrichment of fatty acid metabolism in EAC pathogenesis<br><br>progressive alterations (NE-BE-EAC) with increasing levels of phosphatidylcholine<br><br>changes in phospholipid metabolism during EAC development<br><br>altered significantly phosphatidyl-choline metabolism in ESCC [59]<br><br>Phosphatidylcholine metabolism dysregulated serum of ESCC patients [60]<br><br>significantly different serum levels of metabolites between patients with and without hematological or renal side effects

			higher level of phosphatidylcholines linked to hematological toxicity<br><br>lower serum level of lysophosphatidylcholine linked to nephrotoxicity<br><br>biomarkers for predicting hematological toxicity or nephrotoxicity after neoadjuvant chemoradiotherapy for ESCC [61]<br><br>dysregulated lipid metabolism in ESCC patients<br><br>significant reduction of phosphatidylcholines (PC) in ESCC serum<br><br>phosphatidylcholines as novel biomarkers for ESCC [62]
stomach/gastric (GC)	higher level of the acylcarnitine: C2 in GC cancer vs of first-degree relatives<br>increase C2 in negative H. pylori-GC. [59]	potent oncogenic effect 5-HT receptor 1D (HTR1D) on GC [64]<br><br>FLX-induced apoptosis in AGS (Human gastric cell lines) connected with upregulation of death receptors, ROS generation, and activation of ER stress F [65]<br><br>The expression of 5-HT significantly higher in GC than patients with chronic gastritis ($p < 0.05$).<br><br>5-HT partially co-localized with CgA, in gastric mucosa [66]	phospholipids more abundant in patients with early gastric cancer than in healthy controls<br><br>reduction of the levels of phosphatidylethanolamine (36:2), phosphatidylcholine (32:0), after resected the cancerous lesions<br><br>serum phospholipids pointed as potential biomarkers for the early diagnosis of gastric cancer [67]<br><br>Increase of Phosphatidylcholine (34:3) AUC 0.978 phosphatidylcholine (36:1) in the cancerous than in the paracancerous tissues<br><br>higher in the serum of EGC patients than healthy controls<br><br>reduction of Phosphatidylcholine (32:0), (AUC 1.0) in the cancerous than in the paracancerous tissues<br><br>lower in the serum of EGC patients than healthy controls.

			regions associated with EGC different lipid distributions from the paracancerous regions<br><br>Serum phosphatidylcholine (32:0), (34:3) pointed as potential biomarkers for discriminating between EGC patients and healthy controls [68]<br><br>changes with the phospholipids (mainly PC) between cancer tissue and nonneoplastic mucosa [69]<br><br>Serum levels of phosphatidylcholine (PC) (34:2), PC(34:1), PC(36:4), PC(36:3), and PC(36:2) different and linked with pathophysiological states (colorectal cancer, gastric cancer)<br><br>phospholipids significantly correlated with gender, physiological states, and cancer stages [70]
Liver (HCT)<br><br>hepatocellular carcinoma (HCC)	upregulated carnitine (C2/C0) HCT vs. DNT [67]<br><br>impact of lenvatinib therapy in the carnitine system in patients with hepatocellular carcinoma<br><br>carnitine insufficiency, a common cause of fatigue during the treatment [68]	induction of cell death in hepatocellular carcinoma cell lines, by Fluoxetine (FLX), a selective serotonin inhibitor<br><br>induction of apoptosis in hepatocellular carcinoma cell line Hep3B [88]<br><br>association preoperative intra-platelet serotonin (IP5-HT) with early disease recurrence after liver resection<br>high IP5-HT levels (>134ng/ml IP5-HT) linked with an increased incidence of early tumor recurrence, low IP5-HT levels (<73ng/ml IP5-HT) with a higher rate of morbidity [73]	an increase (2-fold) of phosphatidylcholines (PCs) in hepatic disease patients with HBV replication [81]<br><br>(using of (PET)/x-ray computed tomography (CT) detection of HCC), based on imaging the initial steps of phosphatidylcholine synthesis<br><br>high levels of saturated phosphatidylcholines in most HCC tumors<br><br>fatty acid metabolism connected with for phospholipid membrane synthesis.<br><br>imperfect diagnostic sensitivity of PET/CT identification of lipogenic tumors, connected with metabolic heterogeneity across HCC and a

		regulation of liver cancer cell steatosis, cells survival with serotonin potential promotion of liver carcinogenesis by activation of Notch signaling and autophagy<br><br>enhanced cancer cell proliferation/survival and drug resistance by serotonin.<br><br>upregulation of the expression of lipogenic proteins and increase of steatosis in liver cancer cells with serotonin treatment [74]<br><br>serotonin, essential for HSC survival and activation<br><br>higher synthesis and accumulation in males than in females<br><br>promotion of HCC carcinogenesis in Serotonin-activated HSCs<br><br>increase in serotonin expression synthesis [75]<br><br>significant increase of serotonin in early histological stage of HCC development (rat model, post DENA injection)<br><br>potential role of serotonin as a biomarker used for diagnosis of early stage HCC [76]<br><br>very good serum serotonin discriminating power (sensitivity 100%, specificity 92.3%) in early-stage HCC from cirrhosis<br><br>Serum serotonin level rapid, sensitive, non-	weaker lipogenic phenotype in some tumors [82]<br><br>altered lipid profile in human HCC<br><br>specific lipid metabolic pathways linked with hepatocytes proliferation<br><br>positive correlation of monounsaturated PC with hallmarks of cell proliferation and hepatic carcinogenesis [83]<br><br>relatively poor prognosis, a low concentration of the degradation products of phosphatidylcholine connected with subtype S1 of (HCC) [84]<br><br>disorder of lipid metabolism in (HCC)<br><br>altered phosphatidylcholine (PC) [85]<br><br>phospholipids connected with tumour progression<br><br>metabolic tissue alterations associated with plasmatic modifications<br><br>elevated concentrations of phosphatidylcholine (PC) 16:0/16:1, PC 16:0/16:0 , PC 16:0/18:1 , low concentrations of lysophosphatidylcholine 20:4 HCC patients<br><br>phospholipid profiles related to HCC risk in liver cirrhotic patients<br><br>potential of some phospholipids in predicting HCC patient mortality [86]<br><br>lipids (with phosphatidylcholine) significantly downregulated lipids were in HCC patients compared to chronic liver disease CLD patients, and in HCC tissues compared to nontumor hepatic tissues [87]

		invasive diagnostic biomarker for the detection of early-stage HCC [77]<br><br>selective serotonin reuptake inhibitors (SSRIs) associated with a 34% lower risk of HCC, dose-dependent [78]<br><br>SSRIs, mianserin potentially antitumor activity in HCC [79]<br><br>impact of Serotonin on the tumorigenesis of HCC<br>serotonin - dependable marker for the diagnosis of HCC (AUC 0.942) for screening for HCC in cirrhotic patients with chronic hepatitis CHC [80]	
colon/colorectal (CRC)	cytotoxic effect of C2, C3, C5, C7 on CRC cell lines (HCT116 and SW480) significant reduction in adherent cells and inhibition of HCT116 cell invasion with C2 [85]<br><br>Ffar2 deficiency (human colon cancer cell lines; SW480 and HT29) significantly altered profiles of fatty acid metabolites 9 fatty acid oxidation) and gut microbiome, potentially connected with promotion of colorectal cancer development [86]<br><br>significant reduction of C3, C0, C5:1 before chemotherapy.<br>reduction of C0, and C2 on the second day of chemotherapy (+1 day), increase of C3 [87]	induction of cell death in colon cancer cell line, by Fluoxetine (FLX), a selective serotonin inhibitor<br><br>antiproliferative effects apoptosis-independent by FLX in colon cancer [65]<br><br>plasma serotonin level markedly higher in patients with colorectal cancer,<br><br>compared with healthy controls, and patients with benign colorectal<br><br>higher plasma serotonin associated with nodal metastasis, disease recurrence, increased risk of mortality and shorter recurrence-free and overall survival<br><br>higher stages of colorectal cancer	CRC cell line nonmetastatic (HCT116): increase of phosphatidylcholine (PC) 34 : 1, from exosomes from primary cancer patients and nonmetastatic cells (HCT116) compared with healthy person<br><br>decreased in the metastatic cell line (SW620), and patients, linked to changes in the metastatic cell membrane [96]<br><br>potential roles of lipid metabolism in the early stage of colorectal carcinogenesis (pre-diagnostic plasma metabolome of adenomas, serrated polyps)<br><br>higher levels of C36:3 phosphatidylcholine (PC) plasmalogen associated with lower risk of conventional adenomas [98]<br><br>lipid metabolism associated with colon cancer prognosis and incidence

		associated with higher plasma level of serotonin polymorphisms of one of the serotonin transporters, SLC6A4, independently<br><br>linked with shorter overall survival and<br><br>disease specific survival of patients with colorectal cancer<br><br>association serotonin with intra-tumor angiogenesis<br><br>in colorectal cancer and serotonin deficiency PMID: 34289794<br><br>diverse roles in CRC, promoting the development of CRC<br><br>association with more severe signs and symptoms, expansion [92]<br><br>association of Serotonin (5-hydroxytryptamine, 5-HT) with initiation and progression of CAC colitis-associated cancer (CAC)<br>5-HT/5-HT2B/TGF-β signaling as a critical tumor-suppressing axis during CAC initiation,<br><br>promoting cancer progression in the late-stage of CAC [93]<br><br>reduction of serotonin in colorectal cancer [94]<br><br>5-HT signaling correlated with CRC severity	specific lipid composition critical for CSC (cancer stem cell) maintenance.<br><br>lower phosphatidylcholine (PC; p-18:0/18:1) in CSCs (cancer stem cells) than in bulk cancer cells (BCCs)<br><br>SCD1 inhibition increased specific PC a in CSCs [99]<br><br>alterations in lipid composition in cancer tissues<br><br>compared with normal tissue<br><br>cancer tissues significantly elevated levels of phospholipids<br><br>overexpression of genes associated with fatty acid oxidation, and the synthesis of phospholipids<br><br>reprogramming of lipid metabolism in CRC tissues, for energy production and enhanced synthesis of membrane lipids, necessary for the rapid proliferation of cancer cells [100]<br><br>reduction of PCs in serum of CRC patients compared with healthy one [101]

		inhibition of the self-renewal of colorectal cancer stem cells (CSCs) and therapeutic efficacy against CRC tumors connected with blocking 5-HT signaling in mice [95]	

56. Zang, Q.; Sun, C.; Chu, X.; Li, L.; Gan, W.; Zhao, Z.; Song, Y.; He, J.; Zhang, R.; Abliz, Z. Spatially Resolved Metabolomics Combined with Multicellular Tumor Spheroids to Discover Cancer Tissue Relevant Metabolic Signatures. *Anal Chim Acta* **2021**, *1155*, 338342, doi:10.1016/j.aca.2021.338342.
57. Zemanova, M.; Vecka, M.; Petruželka, L.; Staňková, B.; Žák, A.; Zeman, M. Plasma Phosphatidylcholines Fatty Acids in Men with Squamous Cell Esophageal Cancer: Chemoradiotherapy Improves Abnormal Profile. *Med Sci Monit* **2016**, *22*, 4092–4099, doi:10.12659/msm.896799.
58. Zang, B.; Wang, W.; Wang, Y.; Li, P.; Xia, T.; Liu, X.; Chen, D.; Piao, H.-L.; Qi, H.; Ma, Y. Metabolomic Characterization Reveals ILF2 and ILF3 Affected Metabolic Adaptions in Esophageal Squamous Cell Carcinoma. *Front Mol Biosci* **2021**, *8*, 721990, doi:10.3389/fmolb.2021.721990.
59. Molendijk, J.; Kolka, C.M.; Cairns, H.; Brosda, S.; Mohamed, A.; Shah, A.K.; Brown, I.; Hodson, M.P.; Hennessy, T.; Liu, G.; et al. Elevation of Fatty Acid Desaturase 2 in Esophageal Adenocarcinoma Increases Polyunsaturated Lipids and May Exacerbate Bile Acid-Induced DNA Damage. *Clin Transl Med* **2022**, *12*, e810, doi:10.1002/ctm2.810.
60. Mir, S.A.; Rajagopalan, P.; Jain, A.P.; Khan, A.A.; Datta, K.K.; Mohan, S.V.; Lateef, S.S.; Sahasrabuddhe, N.; Somani, B.L.; Keshava Prasad, T.S.; et al. LC-MS-Based Serum Metabolomic Analysis Reveals Dysregulation of Phosphatidylcholines in Esophageal Squamous Cell Carcinoma. *J Proteomics* **2015**, *127*, 96–102, doi:10.1016/j.jprot.2015.05.013.
61. Nishiumi, S.; Fujigaki, S.; Kobayashi, T.; Kojima, T.; Ito, Y.; Daiko, H.; Kato, K.; Shoji, H.; Kodama, Y.; Honda, K.; et al. Metabolomics-Based Discovery of Serum Biomarkers to Predict the Side-Effects of Neoadjuvant Chemoradiotherapy for Esophageal Squamous Cell Carcinoma. *Anticancer Res* **2019**, *39*, 519–526, doi:10.21873/anticanres.13143.
62. Ma, W.; Wang, S.; Zhang, T.; Zhang, E.Y.; Zhou, L.; Hu, C.; Yu, J.J.; Xu, G. Activation of Choline Kinase Drives Aberrant Choline Metabolism in Esophageal Squamous Cell Carcinomas. *J Pharm Biomed Anal* **2018**, *155*, 148–156, doi:10.1016/j.jpba.2018.03.062.
63. Corona, G.; Cannizzaro, R.; Miolo, G.; Caggiari, L.; De Zorzi, M.; Repetto, O.; Steffan, A.; De Re, V. Use of Metabolomics as a Complementary Omic Approach to Implement Risk Criteria for First-Degree Relatives of Gastric Cancer Patients. *Int J Mol Sci* **2018**, *19*, 750, doi:10.3390/ijms19030750.
64. Jin, X.; Li, H.; Li, B.; Zhang, C.; He, Y. Knockdown and Inhibition of Hydroxytryptamine Receptor 1D Suppress Proliferation and Migration of Gastric Cancer Cells. *Biochem Biophys Res Commun* **2022**, *620*, 143–149, doi:10.1016/j.bbrc.2022.06.088.
65. Khin, P.P.; Po, W.W.; Thein, W.; Sohn, U.D. Apoptotic Effect of Fluoxetine through the Endoplasmic Reticulum Stress Pathway in the Human Gastric Cancer Cell Line AGS. *Naunyn Schmiedebergs Arch Pharmacol* **2020**, *393*, 537–549, doi:10.1007/s00210-019-01739-7.
66. Niu, Q.; Li, L.; Zhang, C.; Qi, C.; He, Q.; Zhu, Y. Expression of 5-HT Relates to Stem Cell Marker LGR5 in Patients with Gastritis and Gastric Cancer. *Dig Dis Sci* **2023**, *68*, 1864–1872, doi:10.1007/s10620-022-07772-6.
67. Zou, L.; Guo, L.; Zhu, C.; Lai, Z.; Li, Z.; Yang, A. Serum Phospholipids Are Potential Biomarkers for the Early Diagnosis of Gastric Cancer. *Clin Chim Acta* **2021**, *519*, 276–284, doi:10.1016/j.cca.2021.05.002.
68. Zou, L.; Wang, L.; Guo, L.; Zhou, W.; Lai, Z.; Zhu, C.; Wu, X.; Li, Z.; Yang, A. Small Molecules as Potential Biomarkers of Early Gastric Cancer: A Mass Spectrometry Imaging Approach. *Clin Chim Acta* **2022**, *534*, 35–42, doi:10.1016/j.cca.2022.06.032.

69. Uehara, T.; Kikuchi, H.; Miyazaki, S.; Iino, I.; Setoguchi, T.; Hiramatsu, Y.; Ohta, M.; Kamiya, K.; Morita, Y.; Tanaka, H.; et al. Overexpression of Lysophosphatidylcholine Acyltransferase 1 and Concomitant Lipid Alterations in Gastric Cancer. *Ann Surg Oncol* **2016**, *23 Suppl 2*, S206-213, doi:10.1245/s10434-015-4459-6.
70. Guo, Y.; Ren, J.; Li, X.; Liu, X.; Liu, N.; Wang, Y.; Li, Z. Simultaneous Quantification of Serum Multi-Phospholipids as Potential Biomarkers for Differentiating Different Pathophysiological States of Lung, Stomach, Intestine, and Pancreas. *J Cancer* **2017**, *8*, 2191–2204, doi:10.7150/jca.19128.
71. Huang, Q.; Tan, Y.; Yin, P.; Ye, G.; Gao, P.; Lu, X.; Wang, H.; Xu, G. Metabolic Characterization of Hepatocellular Carcinoma Using Nontargeted Tissue Metabolomics. *Cancer Res* **2013**, *73*, 4992–5002, doi:10.1158/0008-5472.CAN-13-0308.
72. Okubo, H.; Ando, H.; Ishizuka, K.; Kitagawa, R.; Okubo, S.; Saito, H.; Kokubu, S.; Miyazaki, A.; Ikejima, K.; Shiina, S.; et al. Carnitine Insufficiency Is Associated with Fatigue during Lenvatinib Treatment in Patients with Hepatocellular Carcinoma. *PLoS One* **2020**, *15*, e0229772, doi:10.1371/journal.pone.0229772.
73. Padickakudy, R.; Pereyra, D.; Offensperger, F.; Jonas, P.; Oehlberger, L.; Schwarz, C.; Haegele, S.; Assinger, A.; Brostjan, C.; Gruenberger, T.; et al. Bivalent Role of Intra-Platelet Serotonin in Liver Regeneration and Tumor Recurrence in Humans. *J Hepatol* **2017**, *67*, 1243–1252, doi:10.1016/j.jhep.2017.08.009.
74. Niture, S.; Gyamfi, M.A.; Kedir, H.; Arthur, E.; Ressom, H.; Deep, G.; Kumar, D. Serotonin Induced Hepatic Steatosis Is Associated with Modulation of Autophagy and Notch Signaling Pathway. *Cell Commun Signal* **2018**, *16*, 78, doi:10.1186/s12964-018-0282-6.
75. Yang, Q.; Yan, C.; Yin, C.; Gong, Z. Serotonin Activated Hepatic Stellate Cells Contribute to Sex Disparity in Hepatocellular Carcinoma. *Cell Mol Gastroenterol Hepatol* **2017**, *3*, 484–499, doi:10.1016/j.jcmgh.2017.01.002.
76. Abdel-Hamid, N.M.; Shehata, D.E.; Abdel-Ghany, A.A.; Ragaa, A.; Wahid, A. Serum Serotonin as Unexpected Potential Marker for Staging of Experimental Hepatocellular Carcinoma. *Biomed Pharmacother* **2016**, *83*, 407–411, doi:10.1016/j.biopha.2016.07.005.
77. Mamdouh, F.; Abdel Alem, S.; Abdo, M.; Abdelaal, A.; Salem, A.; Rabiee, A.; Elsisi, O. Serum Serotonin as a Potential Diagnostic Marker for Hepatocellular Carcinoma. *J Interferon Cytokine Res* **2019**, *39*, 780–785, doi:10.1089/jir.2019.0088.
78. Lai, S.-W.; Hwang, B.-F.; Liu, C.-S.; Liao, K.-F. Selective Serotonin Reuptake Inhibitor Use and the Risk of Hepatocellular Carcinoma. *Eur J Clin Pharmacol* **2022**, *78*, 1197–1198, doi:10.1007/s00228-022-03306-1.
79. Huang, Y.-H.; Yeh, C.-T. Anticancer Effects of Antidepressants in Hepatocellular Carcinoma Cells. *Anticancer Res* **2023**, *43*, 1201–1206, doi:10.21873/anticanres.16266.
80. Abdel-Razik, A.; Elhelaly, R.; Elzehery, R.; El-Diasty, A.; Abed, S.; Elhammady, D.; Tawfik, A. Could Serotonin Be a Potential Marker for Hepatocellular Carcinoma? A Prospective Single-Center Observational Study. *Eur J Gastroenterol Hepatol* **2016**, *28*, 599–605, doi:10.1097/MEG.0000000000000569.
81. Yu, L.; Zeng, Z.; Tan, H.; Feng, Q.; Zhou, Q.; Hu, J.; Li, Y.; Wang, J.; Yang, W.; Feng, J.; et al. Significant Metabolic Alterations in Patients with Hepatitis B Virus Replication Observed via Serum Untargeted Metabolomics Shed New Light on Hepatitis B Virus Infection. *J Drug Target* **2022**, *30*, 442–449, doi:10.1080/1061186X.2021.2009841.
82. Kwee, S.A.; Sato, M.M.; Kuang, Y.; Franke, A.; Custer, L.; Miyazaki, K.; Wong, L.L. [18F]Fluorocholine PET/CT Imaging of Liver Cancer: Radiopathologic Correlation with Tissue Phospholipid Profiling. *Mol Imaging Biol* **2017**, *19*, 446–455, doi:10.1007/s11307-016-1020-3.
83. Hall, Z.; Chiarugi, D.; Charidemou, E.; Leslie, J.; Scott, E.; Pellegrinet, L.; Allison, M.; Mocciaro, G.; Anstee, Q.M.; Evan, G.I.; et al. Lipid Remodeling in Hepatocyte Proliferation and Hepatocellular Carcinoma. *Hepatology* **2021**, *73*, 1028–1044, doi:10.1002/hep.31391.
84. Hou, G.; Ding, D.; Tian, T.; Dong, W.; Sun, D.; Liu, G.; Yang, Y.; Zhou, W. Metabolomics-Based Classification Reveals Subtypes of Hepatocellular Carcinoma. *Mol Carcinog* **2022**, *61*, 989–1001, doi:10.1002/mc.23455.

85. Li, Z.; Guan, M.; Lin, Y.; Cui, X.; Zhang, Y.; Zhao, Z.; Zhu, J. Aberrant Lipid Metabolism in Hepatocellular Carcinoma Revealed by Liver Lipidomics. *Int J Mol Sci* **2017**, *18*, 2550, doi:10.3390/ijms18122550.

86. Cotte, A.K.; Cottet, V.; Aires, V.; Mouillot, T.; Rizk, M.; Vinault, S.; Binquet, C.; de Barros, J.-P.P.; Hillon, P.; Delmas, D. Phospholipid Profiles and Hepatocellular Carcinoma Risk and Prognosis in Cirrhotic Patients. *Oncotarget* **2019**, *10*, 2161–2172, doi:10.18632/oncotarget.26738.

87. Ismail, I.T.; Elfert, A.; Helal, M.; Salama, I.; El-Said, H.; Fiehn, O. Remodeling Lipids in the Transition from Chronic Liver Disease to Hepatocellular Carcinoma. *Cancers (Basel)* **2020**, *13*, 88, doi:10.3390/cancers13010088.

88. Ala, M. Tryptophan Metabolites Modulate Inflammatory Bowel Disease and Colorectal Cancer by Affecting Immune System. *Int Rev Immunol* **2022**, *41*, 326–345, doi:10.1080/08830185.2021.1954638.

89. Dahabiyeh, L.A.; Hudaib, F.; Hourani, W.; Darwish, W.; Abu-Irmaileh, B.; Deb, P.K.; Venugopala, K.N.; Mohanlall, V.; Chandrashekharappa, S.; Abu-Dahab, R.; et al. Mass Spectrometry-Based Metabolomics Approach and in Vitro Assays Revealed Promising Role of 2,3-Dihydroquinazolin-4(1H)-One Derivatives against Colorectal Cancer Cell Lines. *Eur J Pharm Sci* **2023**, *182*, 106378, doi:10.1016/j.ejps.2023.106378.

90. Huang, Y.-W.; Lin, C.-W.; Pan, P.; Echeveste, C.E.; Dong, A.; Oshima, K.; Yearsley, M.; Yu, J.; Wang, L.-S. Dysregulated Free Fatty Acid Receptor 2 Exacerbates Colonic Adenoma Formation in Apc Min/+ Mice: Relation to Metabolism and Gut Microbiota Composition. *J Cancer Prev* **2021**, *26*, 32–40, doi:10.15430/JCP.2021.26.1.32.

91. Wang, X.; Wang, J.; Wang, Z.; Wang, Q.; Li, H. Dynamic Monitoring of Plasma Amino Acids and Carnitine during Chemotherapy of Patients with Alimentary Canal Malignancies and Its Clinical Value. *Onco Targets Ther* **2015**, *8*, 1989–1996, doi:10.2147/OTT.S86562.

92. Kannen, V.; Bader, M.; Sakita, J.Y.; Uyemura, S.A.; Squire, J.A. The Dual Role of Serotonin in Colorectal Cancer. *Trends Endocrinol Metab* **2020**, *31*, 611–625, doi:10.1016/j.tem.2020.04.008.

93. Mao, L.; Xin, F.; Ren, J.; Xu, S.; Huang, H.; Zha, X.; Wen, X.; Gu, G.; Yang, G.; Cheng, Y.; et al. 5-HT2B-Mediated Serotonin Activation in Enterocytes Suppresses Colitis-Associated Cancer Initiation and Promotes Cancer Progression. *Theranostics* **2022**, *12*, 3928–3945, doi:10.7150/thno.70762.

94. Shen, Y.; Sun, M.; Zhu, J.; Wei, M.; Li, H.; Zhao, P.; Wang, J.; Li, R.; Tian, L.; Tao, Y.; et al. Tissue Metabolic Profiling Reveals Major Metabolic Alteration in Colorectal Cancer. *Mol Omics* **2021**, *17*, 464–471, doi:10.1039/d1mo00022e.

95. Zhu, P.; Lu, T.; Chen, Z.; Liu, B.; Fan, D.; Li, C.; Wu, J.; He, L.; Zhu, X.; Du, Y.; et al. 5-Hydroxytryptamine Produced by Enteric Serotonergic Neurons Initiates Colorectal Cancer Stem Cell Self-Renewal and Tumorigenesis. *Neuron* **2022**, *110*, 2268-2282.e4, doi:10.1016/j.neuron.2022.04.024.

96. Elmallah, M.I.Y.; Ortega-Deballon, P.; Hermite, L.; Pais-De-Barros, J.-P.; Gobbo, J.; Garrido, C. Lipidomic Profiling of Exosomes from Colorectal Cancer Cells and Patients Reveals Potential Biomarkers. *Mol Oncol* **2022**, *16*, 2710–2718, doi:10.1002/1878-0261.13223.

97. Peng, S.; Li, Y.; Huang, M.; Tang, G.; Xie, Y.; Chen, D.; Hu, Y.; Yu, T.; Cai, J.; Yuan, Z.; et al. Metabolomics Reveals That CAF-Derived Lipids Promote Colorectal Cancer Peritoneal Metastasis by Enhancing Membrane Fluidity. *Int J Biol Sci* **2022**, *18*, 1912–1932, doi:10.7150/ijbs.68484.

98. Hang, D.; Zeleznik, O.A.; Lu, J.; Joshi, A.D.; Wu, K.; Hu, Z.; Shen, H.; Clish, C.B.; Liang, L.; Eliassen, A.H.; et al. Plasma Metabolomic Profiles for Colorectal Cancer Precursors in Women. *Eur J Epidemiol* **2022**, *37*, 413–422, doi:10.1007/s10654-021-00834-5.

99. Choi, S.; Yoo, Y.J.; Kim, H.; Lee, H.; Chung, H.; Nam, M.-H.; Moon, J.-Y.; Lee, H.S.; Yoon, S.; Kim, W.-Y. Clinical and Biochemical Relevance of Monounsaturated Fatty Acid Metabolism Targeting Strategy for Cancer Stem Cell Elimination in Colon Cancer. *Biochem Biophys Res Commun* **2019**, *519*, 100–105, doi:10.1016/j.bbrc.2019.08.137.

100. Mika, A.; Pakiet, A.; Czumaj, A.; Kaczynski, Z.; Liakh, I.; Kobiela, J.; Perdyan, A.; Adrych, K.; Makarewicz, W.; Sledzinski, T. Decreased Triacylglycerol Content and Elevated Contents of Cell Membrane Lipids in Colorectal Cancer Tissue: A Lipidomic Study. *J Clin Med* **2020**, *9*, 1095, doi:10.3390/jcm9041095.

101. Chen, L.; Zhang, C.; Gui, Q.; Chen, Y.; Yang, Y. Ultra-performance Liquid Chromatography Coupled with Quadrupole Time-of-flight Mass Spectrometry-based Metabolic Profiling of Human Serum Prior to and Following Radical Resection of Colorectal Carcinoma. *Mol Med Rep* **2015**, *12*, 6879–6886, doi:10.3892/mmr.2015.4289.
